# Durable biochemical response and safety with oral octreotide capsules in acromegaly

**DOI:** 10.1530/EJE-22-0220

**Published:** 2022-09-29

**Authors:** Susan L Samson, Lisa B Nachtigall, Maria Fleseriu, Mojca Jensterle, Patrick J Manning, Atanaska Elenkova, Mark E Molitch, William H Ludlam, Gary Patou, Asi Haviv, Nienke R Biermasz, Andrea Giustina, Christian J Strasburger, Laurence Kennedy, Shlomo Melmed

**Affiliations:** 1 Department of Medicine and Neurological Surgery, Mayo Clinic, Jacksonville, Florida, USA; 2 Neuroendocrine Unit, Massachusetts General Hospital and Department of Medicine, Harvard Medical School, Boston, Massachusetts, USA; 3 Pituitary Center, Oregon Health & Sciences University, Portland, Oregon, USA; 4 University Medical Centre Ljubljana, Ljubljana, Slovenia; 5 Dunedin Hospital, Dunedin, New Zealand; 6 Department of Endocrinology, Medical University Sofia, USHATE ‘Acad. Ivan Penchev’, Sofia, Bulgaria; 7 Northwestern University Feinberg School of Medicine, Chicago, Illinois, USA; 8 Amryt Pharmaceuticals, Dublin, Ireland; 9 Leiden University Medical Center, Leiden, Netherlands; 10 Institute of Endocrine and Metabolic Sciences, San Raffaele Vita-Salute University, Milan, Italy; 11 Clinical Endocrinology, Charite-Universitätsmedizin, Campus Mitte, Berlin, Germany; 12 Cleveland Clinic Foundation, Cleveland, Ohio, USA; 13 Pituitary Center, Cedars-Sinai Medical Center, Los Angeles, California, USA

## Abstract

**Objective:**

The objective of this study is to report results from the open-label extension (OLE) of the OPTIMAL trial of oral octreotide capsules (OOC) in adults with acromegaly, evaluating the long-term durability of therapeutic response.

**Design:**

The study design is an OLE of a double-blind placebo-controlled (DPC) trial.

**Methods:**

Patients completing the 36-week DPC period on the study drug (OOC or placebo) or meeting predefined withdrawal criteria were eligible for OLE enrollment at 60 mg/day OOC dose, with the option to titrate to 40 or 80 mg/day. The OLE is ongoing; week 48 results are reported.

**Results:**

Forty patients were enrolled in the OLE, 20 each having received OOC or placebo, with 14 and 5 patients completing the DPC period as responders, respectively. Ninety percent of patients completing the DPC period on OOC and 70% of those completing on placebo completed 48 weeks of the OLE. Maintenance of response in the OLE (i.e. insulin-like growth factor I (IGF1) ≤ 1.0 × upper limit of normal (ULN)) was achieved by 92.6% of patients who responded to OOC during the DPC period. Mean IGF1 levels were maintained between the end of the DPC period (0.91 × ULN; 95% CI: 0.784, 1.045) and week 48 of the OLE (0.90 × ULN; 95% CI: 0.750, 1.044) for those completing the DPC period on OOC. OOC safety was consistent with previous findings, with no increased adverse events (AEs) associated with the higher dose and improved gastrointestinal tolerability observed over time.

**Conclusions:**

Patients with acromegaly maintained long-term biochemical response while receiving OOC, with no new AEs observed with prolonged OOC exposure.

## Introduction

Acromegaly is typically caused by a growth hormone (GH)–secreting pituitary adenoma (1, 2, 3). Accordingly, the first-line treatment of acromegaly is usually surgical resection of the pituitary adenoma (4, 5). In patients in whom surgery is not an option or does not achieve complete resection of the hypersecreting tumor, somatostatin receptor ligands (SRLs) are indicated as the first-line medical treatment (2). While they are a mainstay of acromegaly medical treatment, injectable SRLs are associated with a substantial treatment burden, including injection-related adverse events (AEs) resulting from deep tissue injection and breakthrough symptoms at the end of each treatment cycle (6, 7, 8, 9, 10). In addition, a large proportion of patients biochemically controlled (insulin-like growth factor I (IGF1) ≤ 1.3 × upper limit of normal (ULN)) on injectable SRLs report persistent acromegaly symptoms that interfere with their daily lives (11).

Enteric-coated oral octreotide capsules (OOC; MYCAPSSA®, Amryt Pharmaceuticals, Dublin, Ireland), developed as an alternative to injectable SRLs, demonstrated both safety and efficacy in phase 3 clinical studies (12, 13). Results from the double-blind placebo-controlled (DPC) OPTIMAL (Octreotide capsules vs Placebo Treatment In MultinationAL centers; NCT03252353) study showed that OOC may be a well-tolerated and effective alternative to injectable octreotide and lanreotide (13). Mean IGF1 levels in patients treated with OOC were maintained below the ULN after 36 weeks in the DPC period, and the safety profile of OOC capsules was similar to that observed in prior clinical trials of OOC and to the known profile of injectable octreotide and lanreotide, but with no injection-related AE (12, 13). Recently, results of the randomized, open-label MPOWERED (Maintenance of acromegaly Patients with Octreotide capsules compared With injections–Evaluation of REsponse Durability; NCT02685709) study showed that maintenance of biochemical response to OOC was noninferior to injectable SRLs during the 36-week randomized treatment phase (14).

Although prior studies of injectable SRLs indicate a durable response to treatment within the drug class, pivotal trials often assess outcomes at earlier time points. As such, longer-term experience with OOC treatment provides additional valuable information for patients and treating physicians. Here, we report results from the open-label extension (OLE; week 48) of the OPTIMAL trial, describing the long-term durability of biochemical response and safety beyond the initial 36-week core study period.

## Subjects and methods

### Patients and treatment

The OPTIMAL trial consisted of a screening phase, a core DPC period incorporating dose-escalation and fixed-dose phases, and the optional OLE. The study was approved by a local Institutional Review Board or Independent Ethics Committee at each site prior to the initiation of the study. The full list can be found in Supplementary Table 1 (see section on supplementary materials given at the end of this article). Written consent was obtained from each patient after a full explanation of the purpose and nature of all procedures used. Further details for the study and the DPC period results have been previously described (13). In brief, the study enrolled adults with a confirmed diagnosis of acromegaly (pituitary tumor per MRI or pathology), history of active disease (IGF1 ≥ 1.3 × ULN) ≥3 months after the most recent pituitary surgery, and biochemical control on long-acting injectable octreotide LAR or lanreotide (given for ≥6 months and at a stable dose for ≥3 months prior to enrollment). In the DPC period, patients were randomized to receive OOC (40 mg/day starting dose, with subsequent titration to 60 mg/day and then to 80 mg/day per investigator discretion, based on IGF1 and/or symptoms) or placebo.

Patients who participated in the DPC period of OPTIMAL were eligible to enroll in the optional OLE if they completed the DPC period on the study drug (OOC or placebo) or if they met predefined withdrawal criteria and were followed through the end of the 36-week DPC period. All patients entering the OLE received an OOC dose of 60 mg/day, regardless of the dose they were on in the DPC period, with the option to increase dosing to 80 mg/day or decrease to 40 mg/day based on biochemical control, acromegaly signs or symptoms, and safety/tolerability. The pharmacokinetics of OOC enabled a rapid dose titration every 2–4 weeks. All patients were instructed to take OOC 1 h before or 2 h after a meal to maximize bioavailability.

### Assessments and analysis

The OLE of the OPTIMAL trial is ongoing; results presented in this manuscript are an interim analysis with a data cut-off of May 12, 2020, which includes 48 weeks of treatment in the OLE, with all endpoints predefined in the study statistical analysis plan. Populations analyzed for each endpoint can be found in Supplementary Table 2.

All efficacy endpoints in the OLE were exploratory. Key endpoints included i) the proportion of patients who completed 48 weeks of the OLE, specifically out of those who previously received OOC during the DPC period, and ii) the proportion of patients who completed the OLE as responders, out of those who previously received OOC during the DPC period and were classified as responders at the end of the DPC period. The biochemical response was defined by an IGF1 ≤ 1.0 × ULN based on the average of the last two assessments in each period (weeks 34 and 36 for the DPC period and weeks 46 and 48 for the OLE). Patients who discontinued or had mean IGF1 > 1.0 × ULN at week 46/48 of the OLE were classified as nonresponders.

Other efficacy endpoints included changes in IGF1 and GH and shift in IGF1 response categories from the baselines of both the OLE and DPC period to week 48 of the OLE. IGF1 response categories were as follows, based on the average IGF1 level of the last two assessments leading up to each time point of interest (i.e. in the two weeks prior to randomization for DPC baseline, between weeks 34 and 36 in the DPC period for OLE baseline, and between weeks 46 and 48 of the OLE for OLE week 48): responder (IGF1 ≤ 1.0 × ULN), partial responder (IGF1 > 1.0 × ULN and IGF1 < 1.3 × ULN), or nonresponder (IGF1 ≥ 1.3 × ULN). Responder analyses applied a nonresponder imputation approach, in which patients who discontinued treatment during the OLE for any reason were classified as nonresponders. The proportion of patients who were biochemically controlled (i.e. responders) at week 48 of the OLE was explored as an additional efficacy endpoint.

Maintenance of response at the end of the OLE was assessed in the subset of patients who completed the DPC period on the study drug (OOC or placebo). The multiple imputation (MI) approach was used to account for missing IGF1 data. For analyzing GH values, a mixed model for repeated measures (MMRM) was used, including the observed change from baseline to end of OLE treatment and with no imputation for missing data as a dependent variable.

Safety was assessed in all patients enrolled in the OLE and who received ≥1 OOC dose. Key safety endpoints included incidence of treatment-emergent AEs (TEAEs) and serious adverse events (SAEs), changes from baseline in blood chemistry and hematology parameters and vital signs, and incidence of clinically significant findings on abdominal ultrasound during the OLE period.

## Results

### Patient disposition

**Figure 1:**
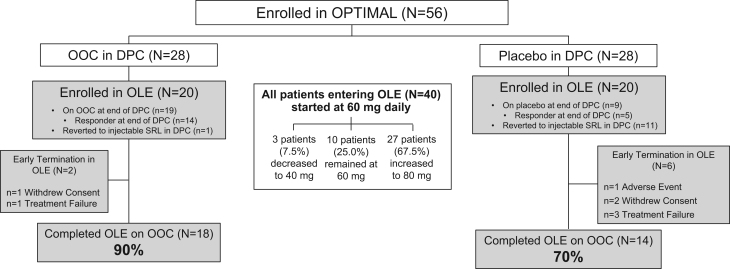
Patient disposition. DPC, double-blind placebo-controlled; OLE, open-label extension; OOC, oral octreotide capsules; SRL, somatostatin receptor ligand.

A total of 40 patients were enrolled in the OLE, representing 71.4% of the total patients in the DPC (71.4% from the OOC group and 71.4% from the placebo group). Of these, 20 were treated with OOC and 20 were treated with placebo during the DPC period, with disposition summarized in Fig. 1. Of the 20 prior OOC recipients entering the OLE (14 responders, 6 nonresponders), 19 completed the DPC period on OOC, with 1 having reverted to their prior injectable SRL. Of the 20 prior placebo recipients entering the OLE (5 responders, 15 nonresponders), 9 completed the DPC period on placebo, with 11 having reverted to their prior injectable SRL.

Thirty-two out of 40 (80%) patients completed the OLE. Eighteen of the 20 prior OOC recipients (90%) completed the OLE on OOC, a key endpoint of the study. Two (10%) prior OOC recipients discontinued the OLE early (1 withdrew consent and 1 listed as treatment failure). Fourteen of the 20 prior placebo recipients (70%) completed the OLE on OOC, and 6 (30%) discontinued the OLE early (1 TEAE, 2 withdrew consent, and 3 treatment failures). Six of the prior OOC recipients and 3 of the prior placebo recipients had missing data owing to coronavirus disease 2019 (COVID-19).

Demographic and baseline characteristics of those entering the OLE are shown in Table 1. Overall, the mean age at screening for the OLE participants was 57.0 years (range, 35–79 years). Most patients were White/Caucasian (87.5%), and 55.0% were male.

**Table 1:** Patient demographic and baseline characteristics.

Category/statistic	OOC dose in the OLE			
	40 mg (*n* = 3)	60 mg (*n* = 10)	80 mg (*n* = 27)	Total (*N* = 40)
Sex, *n* (%)				
Male	2 (66.7)	4 (40.0)	16 (59.3)	22 (55.0)
Female	1 (33.3)	6 (60.0)	11 (40.7)	18 (45.0)
Race, *n* (%)				
Asian	0	1 (10.0)	2 (7.4)	3 (7.5)
Black/African or African American	0	0	1 (3.7)	1 (2.5)
White/Caucasian	3 (100)	9 (90.0)	23 (85.2)	35 (87.5)
Other	0	0	1 (3.7)	1 (2.5)
Age at screening, years, mean (s.d.)	63.0 (5.3)	57.7 (10.1)	56.0 (11.8)	57.0 (11.0)
BMI at screening, kg/m^2^, mean (s.d.)	24.8 (3.1)	31.6 (8.2)	30.9 (5.1)	30.6 (6.0)
Duration of acromegaly, years, *n* (%)				
<10	2 (66.7)	7 (70.0)	14 (51.9)	23 (57.5)
10 to <20	0	2 (20.0)	9 (33.3)	11 (27.5)
≥20	1 (33.3)	1 (10.0)	4 (14.8)	6 (15.0)
Prior acromegaly surgery, *n* (%)	3 (100)	9 (90.0)	25 (92.6)	37 (92.5)
OLE baseline average IGF1, ULN, mean (s.d.)	0.7 (0.20)	0.9 (0.19)	1.0 (0.25)	1.0 (0.25)
OLE baseline average IGF1, ULN, *n* (%)				
≤1.0	3 (100)	8 (80.0)	17 (63.0)	28 (70.0)
>1.0 to <1.3	0	1 (10.0)	5 (18.5)	6 (15.0)
≥1.3	0	1 (10.0)	5 (18.5)	6 (15.0)
OLE baseline GH, ng/mL, mean (s.d.)	1.3 (1.6)	0.5 (0.4)	0.9 (1.0)	0.8 (1.0)
OLE baseline GH, ng/mL, *n* (%)				
<1.0	2 (66.7)	8 (80.0)	19 (70.4)	29 (72.5)
≥1.0 to <2.5	0	2 (20.0)	5 (18.5)	7 (17.5)
≥2.5	1 (33.3)	0	3 (11.1)	4 (10.0)
Prior injectable treatment overall, *n* (%)^a^				
Low	1 (33.3)	4 (40.0)	3 (11.1)	8 (20.0)
Middle	0	2 (20.0)	7 (25.9)	9 (22.5)
High	2 (66.7)	4 (40.0)	17 (63.0)	23 (57.5)
Symptom burden at end of DPC period, n (%)				
≥1	1 (33.3)	4 (40.0)	10 (37.0)	15 (37.5)
≥2	1 (33.3)	1 (10.0)	6 (22.2)	8 (20.0)
≥3	1 (33.3)	0	4 (14.8)	5 (12.5)
Final dose level of study drug (OOC or placebo) during DPC period, mg, *n* (%)				
40	2 (66.7)	3 (30.0)	1 (3.7)	6 (15.0)
60	0	0	1 (3.7)	1 (2.5)
80	0	2 (20.0)	11 (40.7)	13 (32.5)
Completed DPC on SRL treatment, *n* (%)	0	2 (20.0)	10 (37.0)	12 (30.0)

^a^Low dose: octreotide 10 mg every 4 weeks; lanreotide 60 mg every 4 weeks or 120 mg every 8 weeks. Medium dose: octreotide 20 mg every 4 weeks; lanreotide 90 mg every 4 weeks or 120 mg every 6 weeks. High dose: octreotide 30 mg every 4 weeks; lanreotide 120 mg every 4 weeks.

DPC, double-blind placebo-controlled; GH, growth hormone; IGF1, insulin-like growth factor I; OLE, open-label extension; OOC, oral octreotide capsules; SRL, somatostatin receptor ligand; ULN, upper limit of normal.

The mean OOC exposure duration during the OLE was 384.5 days (range, 49–700 days). At OLE week 48, OOC dosing was 40 mg/day for 3 patients (7.5%), 60 mg/day for 10 patients (25%), and 80 mg/day for 27 patients (67.5%). In patients reverting to their prior injectable SRL during the DPC period, the mean time from the last injection during the DPC period to starting OOC in the OLE was 24 days.

### Efficacy endpoints

#### Maintenance of response

Fourteen of the 20 patients who received OOC during the DPC period and enrolled in the OLE completed the DPC period as responders. Of these 14, all 14 completed to week 48 of the OLE. The responder rate at week 48 of the OLE using MI was 92.6% (95% CI: 78.7–100). Thirteen of the 14 (92.9%) patients who completed the DPC on OOC as responders also completed the first year of the OLE as responders, based on their last measurements. Additionally, 5 patients who received placebo during the DPC period and enrolled in the OLE completed the DPC as responders. All 5 (100%) were responders at week 48; therefore, 18/19 (94.7%) patients who were responders in the DPC and enrolled in the OLE maintained their response at week 48.

#### Changes in IGF1 and GH

##### Baseline of OLE to week 48 of OLE

**Figure 2:**
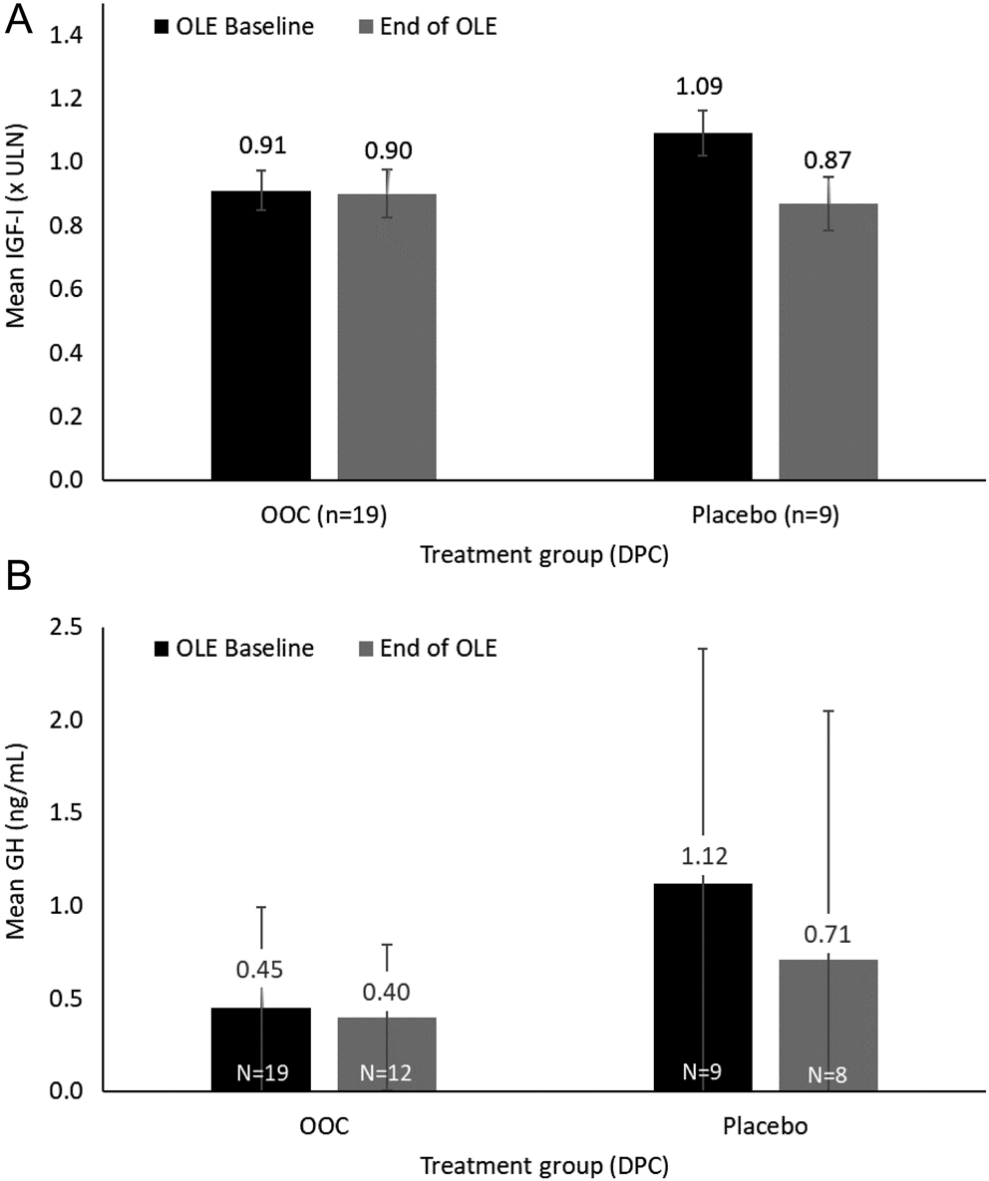
Mean levels of (A) IGF1 and (B) GH from baseline of OLE to week 48 of OLE. Error bars based on s.e. (IGF1) and s.d. (GH). DPC, double-blind placebo-controlled; GH, growth hormone; IGF1, insulin-like growth factor I; OLE, open-label extension; OOC, oral octreotide capsules; ULN, upper limit of normal.

IGF1 levels of patients who completed the DPC period on OOC (*n* = 19) were maintained from the start of the OLE (mean IGF1: 0.91 × ULN; 95% CI: 0.784, 1.045) to week 48 of the OLE (mean IGF1: 0.90 × ULN; 95% CI: 0.750, 1.044; mean change from baseline of 0.004 × ULN; 95% CI: −0.0643, 0.0725, or −0.018 × ULN using MI, 95%, −0.117, 0.081; Fig. 2A). In placebo-randomized patients who completed the DPC period without reversion to their prior injectable SRL (*n* = 9), mean IGF1 improved from 1.09 × ULN (95% CI: 0.928, 1.251) at OLE baseline to 0.87 × ULN (95% CI: 0.702, 1.029) at OLE week 48, a mean change of −0.22 × ULN (95% CI: −0.3986, −0.0308; for MI 95% CI: −0.369, −0.079) with or without MI (Fig. 2A).

In patients who completed the DPC period on OOC, the observed mean change in GH from OLE baseline to week 48 was 0.05 ng/mL; using MMRM, the least-squares mean (LSM) change was −0.11 ng/mL (Fig. 2B). For patients who completed the DPC period on placebo without reversion to their prior injectable SRL, the observed mean change in GH from baseline of the OLE to week 48 was −0.51 ng/mL or −0.37 ng/mL as the LSM using MMRM (Fig. 2B).

##### Baseline of DPC period to week 48 of OLE (84 total weeks)

**Figure 3:**
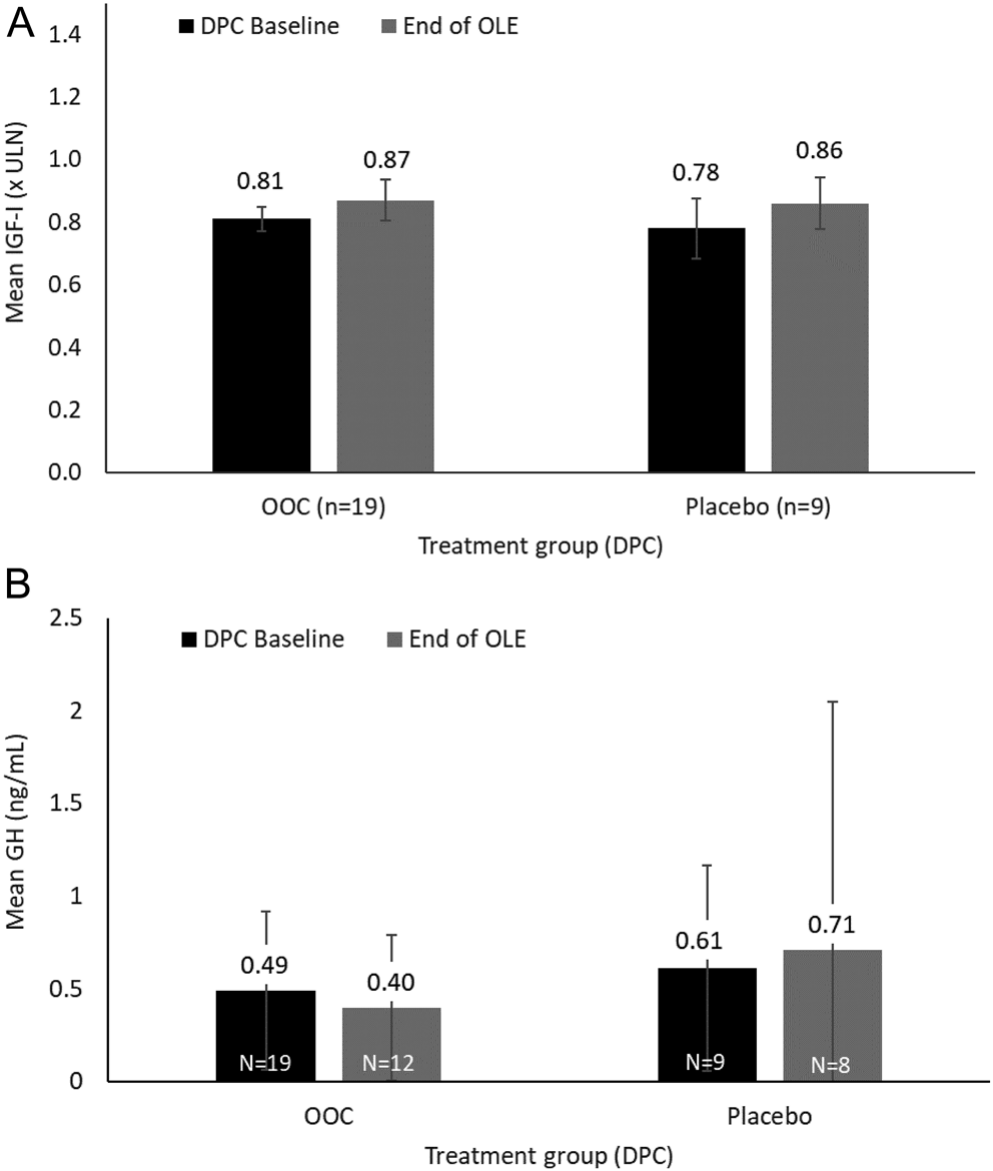
Mean levels of (A) IGF1 and (B) GH from baseline of DPC period to week 48 of OLE. Error bars based on s.e. (IGF1) and s.d. (GH). DPC, double-blind placebo-controlled; GH, growth hormone; IGF1, insulin-like growth factor I; OLE, open-label extension; OOC, oral octreotide capsules; ULN, upper limit of normal.

For patients completing the DPC period on OOC (*n* = 19), the mean change in IGF1 from DPC period baseline (0.81 × ULN; 95% CI: 0.733, 0.894) to OLE week 48 (0.87 × ULN; 95% CI: 0.746, 1.001) was 0.02 × ULN (95% CI: −0.039, 0.087) or 0.06 × ULN (95% CI: −0.050, 0.170) using MI (Fig. 3A). For patients who completed the DPC period on placebo (*n* = 9), the mean change in IGF1 from DPC baseline (0.78 × ULN; 95% CI: 0.554, 0.996) to OLE week 48 (0.86 × ULN; 95% CI: 0.697, 1.017) was 0.09 × ULN (95% CI: 0.005, 0.175) or 0.082 × ULN (95% CI: 0.003, 0.161) using MI (Fig. 3A).

For patients completing the DPC period on OOC, the observed mean GH change between DPC period baseline and OLE week 48 was −0.16 ng/mL, and LSM change using MMRM was −0.11 ng/mL (Fig. 3B). For patients completing the DPC period on placebo, the observed mean and LSM changes were 0.06 and 0.04 ng/mL, respectively (Fig. 3B).

#### Shifting of IGF1 response categories

Fourteen of the 19 patients who completed the DPC period on OOC were responders at OLE baseline; 10 maintained response, one shifted to partial response, and three had missing data at OLE week 48. As reported above, using MI, the responder rate at week 48 of the OLE for those who received OOC during the DPC was 92.6%. Of four patients completing the DPC period on OOC who were partial responders at OLE baseline, one maintained partial response, one discontinued OOC during the OLE, and two had missing data at week 48.

Five of the nine patients who completed the DPC period on placebo were responders at OLE baseline. All five (100%) maintained their response at OLE week 48. One additional patient was a partial responder at OLE baseline and maintained this response at OLE week 48. Two of three prior placebo recipients who were nonresponders at OLE baseline shifted to complete response at OLE week 48 (data were missing for the other patient). Of the nine prior placebo recipients who discontinued placebo (i.e. reverted to their prior injectable SRL) during the DPC period and were responders at OLE baseline, two maintained complete response, one shifted to partial response, and one had missing data at OLE week 48, while five discontinued OOC during the OLE.

### Safety endpoints

All 40 patients who enrolled in the OLE were included in the safety analysis population. During the OLE, 35 patients (87.5%) experienced ≥1 TEAEs, most mild or moderate in severity and considered unrelated to OOC as assessed by the investigator. Sixteen patients (40%) experienced ≥1 TEAEs that were assessed to be OOC related.

The incidence of TEAEs was similar between patients who were previously randomized to placebo (85%) and OOC (90%) during the DPC period. The most common TEAEs overall in the OLE were gastrointestinal (GI) disorders (47.5% of patients), including nausea (20%), diarrhea (12.5%), and vomiting (12.5%). The incidence of GI disorders in the OLE was lower in patients who were randomized to OOC (35%) vs placebo (60%) in the DPC period. Incidences of nausea, diarrhea, and vomiting in the OLE were 10%, 10%, and 5%, respectively, in patients who received OOC in the DPC period, and 30%, 15%, and 20%, respectively, in those who received placebo in the DPC period. Other TEAEs occurring in ≥10% of OLE participants are presented in Table 2.

**Table 2:** Incidence of TEAEs occurring in ≥10% of patients. Data are presented as *n* (%).

	OOC dose in the OLE			
	40 mg (*n* = 3)	60 mg (*n* = 10)	80 mg (*n* = 27)	Total (*n* = 40)
Patients with ≥1 TEAE	2 (66.7)	10 (100)	23 (85.2)	35 (87.5)
Nausea	0	2 (20.0)	6 (22.2)	8 (20.0)
Diarrhea	0	0	5 (18.5)	5 (12.5)
Vomiting	0	2 (20.0)	3 (11.1)	5 (12.5)
Fatigue	0	1 (10.0)	3 (11.1)	4 (10.0)
Peripheral swelling	0	0	4 (14.8)	4 (10.0)
Nasopharyngitis	2 (66.7)	2 (20.0)	2 (7.4)	6 (15.0)
Urinary tract infection	0	2 (20.0)	3 (11.1)	5 (12.5)
Arthralgia	0	3 (30.0)	5 (18.5)	8 (20.0)
Blood glucose increased	0	2 (20.0)	4 (14.8)	6 (15.0)
Carpal tunnel syndrome	0	4 (40.0)	1 (3.7)	5 (12.5)
Dizziness	0	2 (20.0)	2 (7.4)	4 (10.0)
Headache	0	0	4 (14.8)	4 (10.0)
Hyperhidrosis	0	1 (10.0)	4 (14.8)	5 (12.5)

OLE, open-label extension; OOC, oral octreotide capsules; TEAE, treatment-emergent adverse event.

In a* post hoc* analysis, TEAE incidence was 57.9% among patients on 60 mg/day in the OLE (i.e. those on placebo during the DPC period and not previously exposed to OOC), compared with 96.4% in patients initiating OOC 40 mg/day in the DPC period. The percentage of patients with TEAEs deemed to be treatment-related was 31.6% in those on 60 mg/day and 53.6% in those on 40 mg/day. GI disorders were the most frequent TEAE in this* post hoc* analysis at 47.4% in those on 60 mg/day and 57.1% in those on 40 mg/day.

Five patients (12.5%) experienced a total of six SAEs during the OLE (individual events of complete atrioventricular block, coronary artery disease, cystic lymphangioma, amaurosis fugax, dehydration, and acute kidney injury). None of the SAEs were considered by the investigators to be possibly or probably related to OOC. There were no deaths reported during the OLE. One patient who received placebo during the DPC period discontinued OLE treatment owing to a TEAE, a mild headache considered unrelated to OOC. No clinically meaningful changes were noted with respect to laboratory safety parameters, vital signs, or gallbladder ultrasounds.

## Discussion

OOCs, recently approved by the US Food and Drug Administration, are a treatment option for patients with acromegaly who have previously responded to injectable octreotide LAR or lanreotide. OOC safety and efficacy during the 36-week DPC period of the phase 3 OPTIMAL pivotal study were reported previously; of note, 90% of patients receiving OOC at the end of the DPC period chose to enroll in the OLE (13). Similar to long-term treatment with injectable SRLs (15), OOCs do not appear to exhibit tachyphylaxis in patients with acromegaly; however, until now, data relating to the long-term persistence of acromegaly control with OOC beyond 13 months (12) were lacking.

The analysis reported here describes the long-term persistence and durability of OOC treatment response and safety for patients who completed 48 weeks of an OLE after completing the 36-week DPC period. Specifically, patients who completed the DPC period on OOC maintained biochemical control, as indicated by mean IGF1 ≤ 1.0 × ULN, through week 48 of the OLE. All patients who enrolled into the OLE as OOC responders completed up to week 48 of the OLE, with 92.6% maintaining OOC response at the end of this period using MI, supporting previous results showing maintenance of biochemical response to OOC in 85% of patients (12).

Crossover to OOC in the OLE appeared to benefit patients who had been receiving placebo. Among patients who completed the DPC period on placebo (*n* = 9), the mean IGF1 decreased from 1.09 × ULN (95% CI: 0.928, 1.251) to within normal limits (0.87 × ULN; 95% CI: 0.702, 1.029) by week 48 of the OLE, while receiving OOC. All patients who completed the DPC period on placebo as complete (*n* = 5) or partial (*n* = 1) responders maintained their response categories at week 48 of the OLE. Two of the three prior placebo recipients who completed the DPC period as nonresponders shifted to complete response by week 48 of the OLE while on OOC.

As understood from experience with using current injectable SRLs, dose uptitration is key to optimizing octreotide and lanreotide efficacy (16, 17, 18). To simplify the dose titration and implement a rapid dose adjustment scheme (i.e. achieving a target individual therapeutic dose with only one dose adjustment, either by up-titration or down-titration), the mid-dose of 60 mg/day was used as the initial dose for all patients in the OLE. This has also been commented on in recent international guidelines (18). The safety profile was similar to that observed for the original dose titration scheme observed during the DPC period (13), in which patients were started at the lowest dose (40 mg/day) and titrated up to the highest dose with 2 dose adjustments. As shown in a* post hoc* analysis, TEAEs were similar in nature and were no more frequent in OOC-naïve patients on 60 mg/day in the OLE than in patients initiating OOC at the lowest dose (40 mg/day) in the DPC period. While differences in the likelihood of TEAE reporting across sequential phases of a lengthy trial may have contributed to lower reported TEAE rates in the OLE, the results of our analysis are consistent with previous analyses showing no dose-related AEs occurring with OOC (12).

The incidence of GI TEAEs in the OLE was lower among patients who received OOC vs placebo in the DPC period, supporting the notion that tolerability improves in patients receiving prolonged treatment relative to OOC-naïve patients. The OOC safety profile is consistent with that of injectable SRLs and known disease burden but without injection-associated AEs. Hyperglycemic episodes were rarely observed during the study, consistent with results shown with injectable first-generation SRLs (19), even when used in high doses (20). Since the adverse diabetogenic effect of some SRLs is clinically meaningful (21) and may impact morbidity in acromegaly, the rarity of hyperglycemia in this study, along with the other safety findings, supports long-term clinical use of OOC.

Limitations of this OLE study and analysis include susceptibility to inherent selection bias relating to the inclusion of participants who elected to not only participate in the core study to possibly receive OOC but to also continue into the OLE because they are either benefitting from treatment with OOC or wish to receive OOC if they were on placebo in the DPC (22). On a broader scale, owing to the impact of COVID-19, several missing data points during the OLE required application of MI to account for missing information. Furthermore, clinical information, including comorbidities and tumor dimensions (23), was not collected. Although the sample size was also relatively small, the study allowed for long-term assessment of OOC safety and efficacy, with 90% of OOC recipients in the DPC period who continued into the OLE completing 48 weeks of the OLE on OOC. Another limitation that is not specific to only this study is the variability of IGF1 normalization in response to SRLs. A previous systematic review found that, in the context of clinical trials, the rate of control of IGF1 among medically naïve patients with long-acting lanreotide or octreotide ranged from 27% to 62% (average 44%) at 11– to 12 months of therapy (24). The highest control rate of IGF1 achieved in the studies analyzed was 70% but with a longer duration of therapy (108 months, median 48 months) (25). In a separate meta-analysis of 90 cohorts from 79 publications, the overall efficacy rate was 55% for IGF1 normalization (26). Lessons from these clinical trials, as well as growing clinical experience with OOC, will continue to help improve our understanding of the efficacy of OOC compared to iSRLs.

Overall, our data suggest that OOC may have a favorable risk-benefit profile in the management of patients with acromegaly currently controlled by injectable octreotide LAR or lanreotide, supporting the use of OOC in endocrinology practice. Long-term biochemical response is important in preventing the progression of acromegaly comorbidities (4, 27), which, if not adequately controlled, may lead to diminished quality of life and increased mortality (4). Availability of an oral long-term therapeutic option offers the potential additional advantage of patient convenience, reducing both the burden of regular injections as well as the frequency of required in-person clinic visits, particularly when access to health care is compromised, as occurred during the COVID-19 pandemic (28, 29).

Results from the OLE of the OPTIMAL trial demonstrate that prolonged OOC treatment results in maintenance of response with no new emerging safety concerns, improved GI tolerability over time, and provide reassuring insights into the safety of a higher OOC dose. Integration of OOC in the treatment algorithm for suitable patients has the potential to facilitate individually tailored acromegaly management.

## Supplementary Material

Supplementary Material

## Declaration of interest

S L Samson is an advisory board member for Novartis and Amryt, a grant recipient from Novartis, and a research investigator at Amryt, Corcept, Novartis, and OPKO. L B Nachtigall is an advisory board member at Pfizer and receives a consulting fee, a grant recipient from Amryt and Ipsen, a speaker at Ipsen, and a research investigator at Amryt. M Fleseriu receives consulting fees from Amryt, Ipsen, Ionis, Recordati, and Pfizer; is a research investigator at Amryt, Crinetics, Ionis, and Recordati; and is a deputy editor at EJE. She was not involved with the peer review process of this paper. M Jensterle is an advisory board member and speaker at Amgen, Novartis, and Pfizer. P J Manning has nothing to disclose. A Elenkova is a grant recipient from SI, Novartis, and Pfizer. M E Molitch received consulting fees from Corcept, Janssen, Merck, NovoNordisk, Pfizer, and Novartis is a grant recipient from Amryt, Crinetics, and Ionis; and a research investigator at Bayer. W H Ludlam was an employee at Amryt Pharma. G Patou is an employee and stock owner at Amryt Pharma. A Haviv is an employee at Amryt Pharma. N R Biermasz has nothing to disclose. A Giustina is an advisory board member at Amryt; receives a consulting fee from Novo, Recordati, Ipsen, and Pfizer; and a grant recipient of Novartis, Ipsen, and Pfizer. C J Strasburger is an advisory board member at Sandoz, Ipsen, and Recordati; receives consulting fees from Ascendis, Chiasma, NovoNordisk, Merck, Sandoz, and Recordati; and is a speaker for Ipsen, NovoNordisk, and HRA-Pharma. L Kennedy received lecture fees from Pfizer. S Melmed is an advisory board member for Ionis and Crinetics; receives a consulting fee from Ipsen; and is a grant recipient from Pfizer.

## Funding

This work did not receive any specific grant from any funding agency in the public, commercial, or not-for-profit sector.

## Author contribution statement

G Patou, A Haviv, M E Molitch, S L Samson, M Fleseriu, W H Ludlam, C J Strasburger, and S Melmed contributed to the conceptualization. A Haviv contributed to data curation and formal analysis. M E Molitch and M Fleseriu contributed to investigation. G Patou, A Haviv, W H Ludlam, and C J Strasburger contributed to the methodology. A Haviv and W H Ludlam contributed to project administration, resources, validation, and visualization. G Patou, A Haviv, M E Molitch, S L Samson, M Fleseriu, W H Ludlam, and S Melmed contributed to supervision. A Haviv, S L Samson, and W H Ludlam contributed to writing the original draft. All authors contributed to reviewing and editing.
