# Supplementary material for: Durable biochemical response and safety with oral octreotide capsules in acromegaly
Source: Eur J Endocrinol. 2022 Sep 29;187(6):733–41. doi: 10.1530/EJE-22-0220 (PMC9641789; doi:10.1530/EJE-22-0220)
Supplement: Supplementary Material [file supplementary_material.pdf]

Supplementary Table 1. List of Institutional Review Boards/Independent Ethics Committees

Site	Independent Ethics Committee
0103	University of Southern California, Institutional Review Board
0104	OHSU Research Integrity
0105	Columbia University Medical Center IRB
0106	Western Institutional Review Board (WIRB)<br><br>Memorial Sloan-Kettering Cancer Center Institutional Review Board/Privacy Board (IRB/PB)
0107	Western Institutional Review Board (WIRB)
0108	UCLA Office of the Human Research Protection Program
0110	Research Compliance Office, Stanford University
0111	Western Institutional Review Board (WIRB)
0112	Cedars-Sinai Medical Center Institutional Review Board, Office of Research Compliance and Quality Improvement
0114	Western Institutional Review Board (WIRB)
0116	Johns Hopkins Institutional Review Boards
0117	Western Institutional Review Board (WIRB)
0119	Washington University Human Research Protection Office
0120	University of Utah Institutional Review Board
0122	Western Institutional Review Board (WIRB)
0124	Western Institutional Review Board (WIRB)
0125	Cook County Health & Hospitals System IRB
0128	Cleveland Clinic IRB
0129	Western Institutional Review Board (WIRB)
0301<br>0303	Ethics Committee of the Medicinal Faculty of Ludwig-Maximilians-Universität
0402<br>0403<br>0404	Egeszsegligyi Tudomanyos Tanacs - Klinikai Farmakologiai Etikai Bizottsag
0501	Rabin Ethics Committee
0502	Sourasky Ethics Committee
0503	Hadassah Ethics Committee
0601	Comitato Etico Regione Toscana - Area Vasta Nord Ovest
0604	Comitato Etico lndipendente Azienda Ospedaliero Universitaria di Cagliari
0801<br>0802	Commissie Medische Ethiek Leiden Medical University Center
0901<br>0902<br>0903<br>0904<br>0906	Komisja Bioetyczna przy Uniwersytecie Medycznym we Wroclawiu
1401<br>1402<br>1405	East Midlands - Leicester Central Research Ethics Committee
1605	McGill University Health Centre Ethic Board
1606	Providence Healthcare Research Institute
1607	Western University-Research Western
1701	De Videnskabsetiske Komitéer for region Midtjylland

1702	
1801	Regional Ethics Committee in Gothenburg
1901<br>1903<br>1904<br>1905	Hacettepe University Medical Faculty Clinical Trial Ethics Committee
2001<br>2002<br>2003<br>2004<br>2005<br>2006	St Vincent's Hospital (Melbourne) Human Research Ethics Committee
2201	Ethics Committee for Multicenter Trials – Bulgarian Drug Agency
2502	Ethics Committee for Clinical Research at Development Society of Pauls Stradins Clinical University Hospital
2601<br>2602	Health and Disability Ethics Committee – New Zealand Ministry of Health
2701	Republic of Slovenia National Medical Ethics Committee

Supplementary Table 2. List of Endpoints and Populations Assessed

Endpoint	Populations Analyzed	Pre-specified or Post hoc
Proportion of patients who completed the week 48 OLE	OLE-OOC	Pre-specified
	OLE-PBO	Post hoc
Proportion of patients who completed the week 48 OLE as responders	OLE-OOC-RESP	Pre-specified
	OLE-PBO-RESP	Post hoc
Change in IGF-I from the baseline of the DPC to week 48 of the OLE	OLE-OOC-COMP	Post hoc
	OLE-PBO-COMP	Post hoc
Change in IGF-I from the baseline of the OLE to week 48 of the OLE	OLE-OOC-COMP	Post hoc
	OLE-PBO-COMP	Post hoc
Change in GH from the baseline of the DPC to week 48 of the OLE	OLE-OOC-COMP	Post hoc
	OLE-PBO-COMP	Post hoc
Change in GH from the baseline of the OLE to week 48 of the OLE	OLE-OOC-COMP	Post hoc
	OLE-PBO-COMP	Post hoc
Shift in IGF-I response categories from the baseline of the DPC period to week 48 of the OLE	OLE-OOC-COMP	Post hoc
	OLE-PBO-COMP	Post hoc
Shift in IGF-I response categories from the baseline of the OLE period to week 48 of the OLE	OLE-OOC-COMP	Post hoc
	OLE-PBO-COMP	Post hoc
Safety End points	OLE	Pre-specified
Incidence of TEAEs	OLE-PBO	Post hoc
	OLE-OOC	Post hoc
TEAE by OOC dose	OLE-OOC – 60 mg	Post hoc
	DPC-OOC – 40 mg	Post hoc

<u>Populations:</u>

- OLE, all patients in the OLE
- OLE-OOC, patients who received OOC in the DPC who enrolled in the OLE
- OLE-OOC-RESP, patients enrolled in OLE and received OOC in the DPC and were responders at end of DPC
- OLE-OOC-COMP, patients enrolled in the OLE and received OOC in the DPC who did not revert to iSRLs
- OLE-PBO, patients who received placebo in the DPC and enrolled in the OLE
- OLE-PBO-RESP, patients enrolled in the OLE and received placebo in the DPC and were responders at end of DPC
- OLE-PBO-COMP, patients enrolled in the OLE and received placebo in the DPC who did not revert to iSRLs
